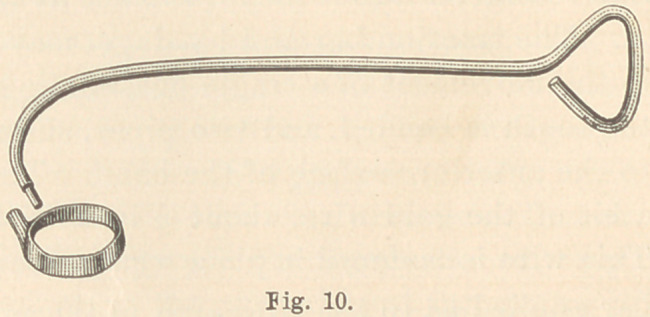# Regulating Appliances

**Published:** 1889-06

**Authors:** Edward H. Angle

**Affiliations:** Minneapolis, Minn.


### REGULATING APPLIANCES.

BY EDWARD H. ANGLE, D.D.S., MINNEAPOLIS, MINN.

Among the many forms of dental irregularities, that form characterized by excessive prominence of the superior oral teeth illustrated in Fig. 1, is one, one which has always been regarded as difficult to treat, and the results have usually been far from satisfactory. It would be interesting to know the percentage of such irregularities; but upon this point our literature is very meagre. Judging from the number occurring in my own practice, and from the large number of models received from other dentists, I am inclined to believe that the percentage is far larger than is usually supposed.

Fig. 1.

It would be interesting to consider the causes that contribute to this deformity; but which, owing to lack of time, must be deferred to the future, it being my purpose now only to describe an appliance for the treatment of these cases.

I think all those who have had experience in treating dental irregularities will agree with me that, where so many teeth are to be drawn back, the molar teeth are insufficient for anchorage. Owing to this insufficiency, the usual result is that the molar teeth are tipped forward and faulty occlusion established, without accomplishing the desired result.

Figure 2.

The value of the occipital bandage, as a means of anchorage is, I believe, becoming more and more appreciated, and is especially applicable to this class of cases. I am using the appliance herein described, in my sixteenth case, and I consider it much more satisfactory than any of the few devices which are described in our literature on this subject. This is shown in part, in Figure 2.

It is made and applied as follows :

The first molars are carefully and accurately banded. These bands may be made of gold or platinum ; but what I regard much better than either, on account of its tensile strength, is German silver, rolled to No. 36 guage, shown in F in Figure 3.

Little pipes about five-eighths of an inch in length are soldered on the side of the arch to the bands. A wire of *hard drawn* platinized gold, about No. 19 gauge, and long enough to encircle the arch is now carefully bent to conform to the shape of the arch, if the arch be correct in form ; but if it be contracted or the teeth irregular, no attention is paid to the form of the existing arch, but an *ideal arch* for the case is made by bending the wire arch to the exact shape to which we wish the teeth in the arch to arrange themselves when the operation is completed.

The ends of this ideal arch are now slipped into the pipes on the molars. The anterior part of the arch is kept from sliding up and impinging upon the gum, by resting in suitable niches formed in the delicate bands encircling, and cemented to the central incisors.

It will also be seen by referring to this cut that two small pipes or collars have been slipped on the wire arch, and are shown in the region of the cuspids. (Also shown at R, Fig. 3.)

These collars are prevented from slipping by being previously soldered into place, care being taken to use *soft solder*, that the temper may not be drawn from the wire arch. The collars are for

Figure 3.

This cut illustrates the full set of my regulating and retaining appliances. Not all the pieces are used, however, in the appliances described in this article.

the purpose of preventing the silk ligatures shown in the cut from slipping backward on the wire. These silk ligatures serve to attach delicate rubber ligatures, which have been hooked over the ends of the little pipes on the anchor teeth, and are represented by dark lines in cuts 2 and 4. The use of these rubber ligatures will be explained further on. Fig. 4 represents a traction bar used in conveying the force from the occipital bandage and distributing it to the wire arch.

Figure 4.

A spur about three-eighths of an inch in length will be seen in the center of this bar, it has a deep niche in one end, which when in position, is placed in contact with the wire arch, at a point between the central incisors. Heavy rubber bands are now attached to the occipital bandage, the other ends being hooked over the end of the traction bar. Shown in position in Fig. 5.

If the reader is familiar with the appliance so far described, it will be seen that the force received from the occipital bandage, is distributed to the wire arch practically through a ball and socket-joint, as the ends of the traction bar may be moved in any direction without interfering with the pressure from the bandage.

The main feature, however, is that in consequence of this freedom of motion, any jar or shock upon the traction bar, will not be transmitted to the tender teeth. As the bandage and bar are to be worn only at night, shocks from contact with the pillow would be very liable to occur, and be very painful were it not for this ball and socket-joint preventing the jar from being transmitted. This is a point of advantage, which I think all will appreciate, and one possessed by no other device with which I am familiar; as the usual method is rigidly to attach the traction bar to a swaged or vulcanized cap covering, and firmly resting against *all* the teeth to be removed.

As the heavy rubber ligatures of the bandage act during the night only, provision must be made to hold through the day what is gained at night. This is accomplished by the delicate rubber ligatures already described.

It will be seen that as the wire arch is forced back through the tubes, the delicate ligatures will prevent it from springing forward, thus suppporting and effectually preventing the loosened teeth from springing back and interfering with the healing process. This is a principle of much importance, and should be carefully observed in the movement of all teeth. And when disregarded, as is too often the case, excessive soreness and much suffering is the inevitable result.

Another advantage of this device, is that not only is prominence of the teeth reduced, but teeth that are irregular are gradually forced to take regular positions, and conform to the shape of the ideal arch, something impossible with devices having fixed caps of vulcanite or gold. Another advantage: if the arch needs expanding, which is frequently necessary in these cases, it may easily be accomplished at the same time the teeth are being moved backward by tightly lacing to the wire arch such teeth as need to be moved outward.

As for the bandage proper, I greatly prefer the common silk travelling cap, shown in the engraving, or the knit jersey cap, to the contrivance usually used for this purpose, as these fit the head snugly, thereby distributing the force exerted by the strong ligatures over more surface, and are consequently more easily worn. Two ligatures should be attached to the cap, one above the ear, and one below, as shown in Fig. 5. If the bands be of equal width, the force will be exerted in the direction of the meatus of the ear.

Figure 5.

This is the point to which the force in most cases should be directed; in some cases, however, the teeth should be compressed in their sockets as well as drawn backwards. This is easily accomplished by dispensing with the ligature below the ear, using the upper ligature only, but of double strength, attaching it at a point on the cap as far forwards as is desired. Again, if elongation of the teeth be necessary, as they are moved backward, the lower ligature only is used, dispensing with the upper. Thus it will be seen, that we have complete control of the moving teeth. After the teeth have become moved into the desired position, they are effectually retained by the wire arch; keeping the same by passing a delicate drill through the pipes on the anchor teeth, and inserting neatly fitting pins into the holes thus made. The head-gear and delicate ligatures are then, of course, dispensed with; the patient will wear this retainer without inconvenience as long as desired.

The traction bar and bandage may also be used to advantage in the movement of a single outstanding incisor. For this purpose the tooth is banded, and two pipes, shown at R, Fig. 3, are soldered to the anterior surface of the band. Through these pipes is passed a bit of the gold wire, about $\frac{1}{4}$ inch in length, shown at G, Fig. 3. This wire is fastened in place with solder, and the traction bar applied as to the wire arch in the previous case. This little device is shown in Fig. 6.

Fig. 6.

The bandage and bar may be used to assist in the double rotation of the central incisors. Fig. 7 shows a case of this kind, where the centrals stood considerably turned across the line of the arch. The two teeth were banded, and two of the pipes, shown at R, Fig. 3, were soldered to these bands, one horizontal and the other perpendicular. A piece of No. 13, *German* piano wire—heavier need not be used—is bent in the form of a hook and placed in position as shown in Fig. 8.

Fig. 7.

Fig. 8.

The tendency of the wire to strenghten itself, as shown by the dotted lines in Fig. 7, will, in a short time, rotate both teeth. It may be necessary to remove and straighten the wire to give it enough spring to do all the work. This simple device will itself, in most instances, speedily accomplish the desired result; but in some instances, where there is much lateral pressuer from the other teeth, or where the external plate of the alveolus is very thin, there may be a tendency for the teeth to spring outward as they rotate. In this case, the bandage and bar may be applied for a few nights, and will effectually prevent any undue prominence of the teeth, which are being rotated.

Fig. 9.

This apparatus may be used in almost the same way when single rotation is being accomplished, as shown in Fig. 9, and there is any tendency of the rotating tooth to spring forward.

The bandage, with a modification of the bar, as shown in Fig. 10, may be used in drawing back that very difficult tooth to move, the cuspid. The cuspid is banded, and to the band the pipe shown at D, Fig. 3, is rigidly soldered. A wire bent in the form of a hook, as shown

Fig. 10.

in Fig. 10, is fitted snugly into this tube, and the heavy elastic is fastened from this hook to the cap on one side only. The snug fit of the hook into the pipe will not permit of any rotation, as the tooth is drawn back, and the tooth is easily and quickly tipped back into position.

Other appliances of the bandage and bar might be given, but will suggest themselves to any one using this valuable appliance.

## Figures and Tables

**Fig. 1. f1:**
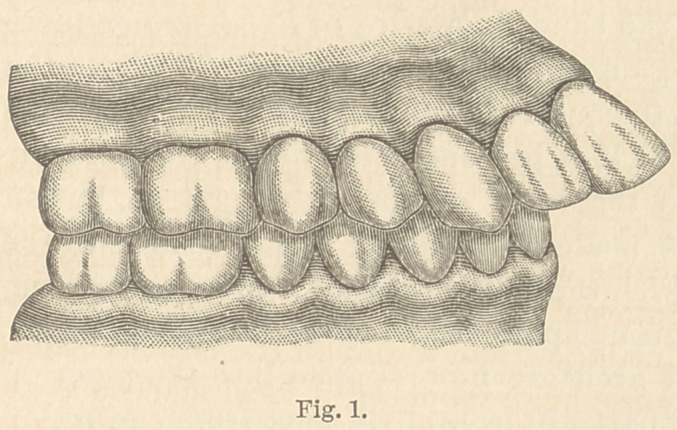


**Figure 2. f2:**
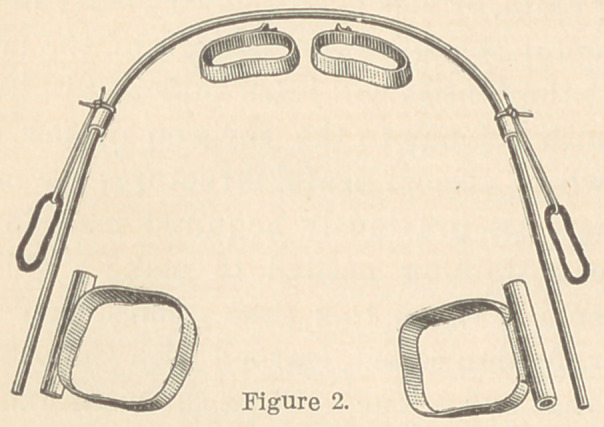


**Figure 3. f3:**
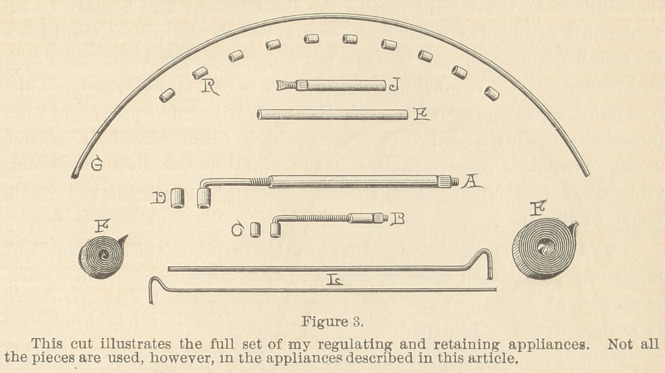


**Figure 4. f4:**
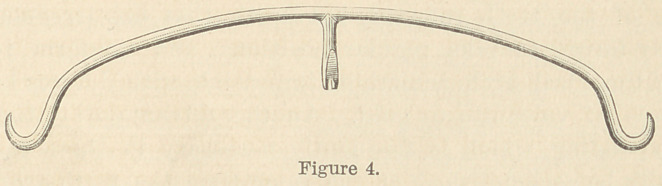


**Figure 5. f5:**
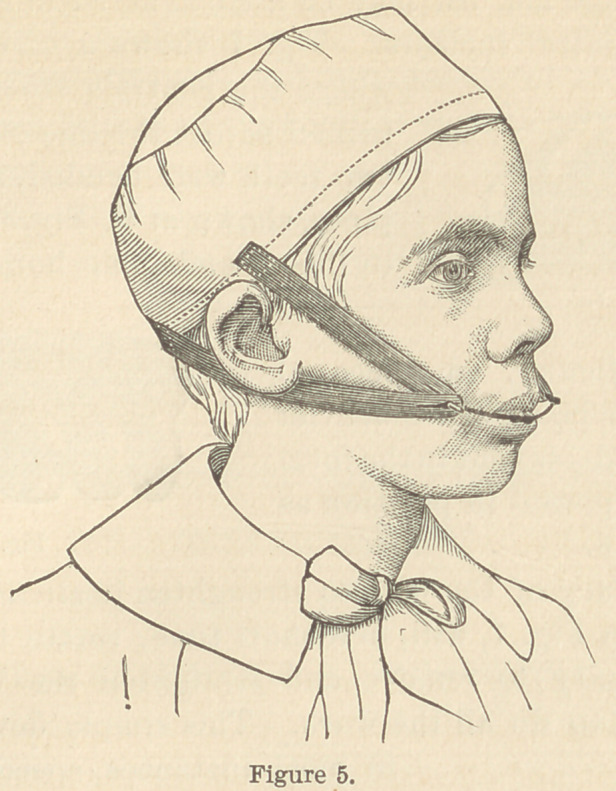


**Fig. 6. f6:**
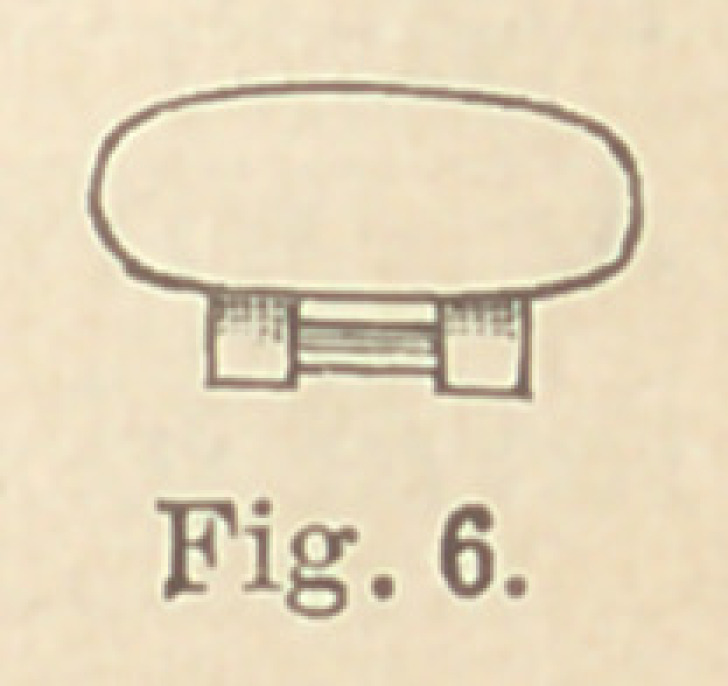


**Fig. 7. f7:**
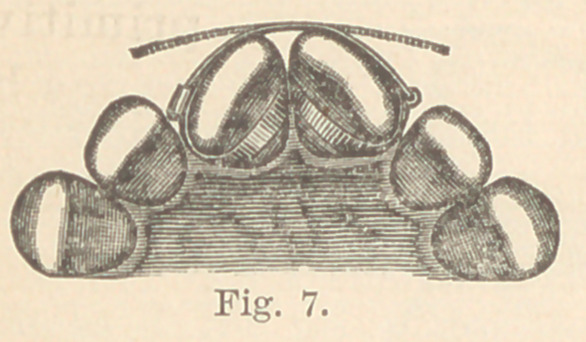


**Fig. 8. f8:**
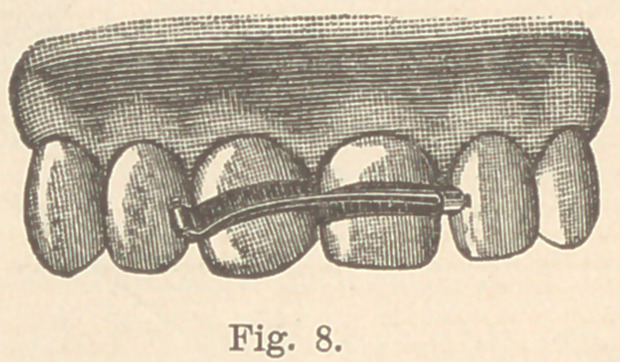


**Fig. 9. f9:**
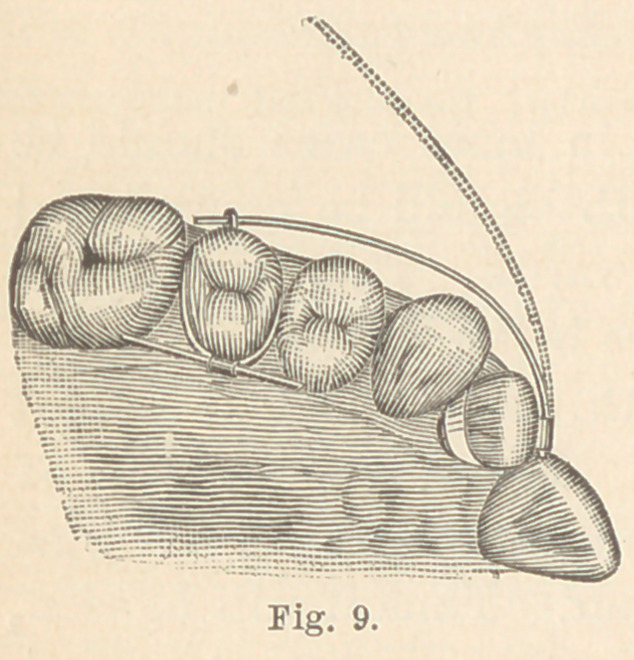


**Fig. 10. f10:**